# Pyelitis in Children

**Published:** 1909-01-30

**Authors:** J. H. Thursfield

**Affiliations:** Assistant Physician to the Hospital for Sick Children, Great Ormond Street, and to the Metropolitan Hospital


### Hospital Clinics.

### PYELITIS IN CHILDREN.

By J. H. THURSFIELD, M.D., F.R.C.P.; Assistant Physician to the Hospital for Sick Children, Great Ormond Street, and to the Metropolitan Hospital.

(A Lecture delivered at Great Ormond Street.)

The subject I have chosen to speak about this afternoon is a disease which is not very common; but I believe it is fairly often overlooked, and so becomes of importance. As you know, there are many febrile diseases of infants which are never fully diagnosed, but are called "febricula," some of which, I am convinced, are due to temporary affections of the urinary tract.

For a variety of reasons the presence of pus in the urine of infants and young children is frequently overlooked. The difficulty of obtaining it in the case of infants and the absence of symptoms definitely pointing to a lesion of the urinary tract lead to a neglect of the examination. Where this is carried out in a routine fashion, pus cells in limited numbers are often found in the urine of patients who present no symptoms of disease of the urinary passages, especially when for any reason the patient is below his usual standard of health. Thus in anæmic boys and girls of school age centrifugalisation of a urine that looks perfectly clear will usually reveal the presence of a few pus corpuscles. The same fact is also observed in the urine of adults who are suffering from a general disturbance of health, and in convalescence from any severe illness. But it is a safe rule to disregard the phenomenon and to attribute to it no pathological significance, unless after centrifugalisation there is visible to the naked eye a deposit which, under the microscope, is found to consist mainly of pus cells.

### The Significance of Pyuria in Children.

When, however, the deposit in the tube is visible to the unaided eye, the question immediately arises as to how far this is of diagnostic significance; whether it is merely the result of some temporary irritation of the urinary passages, or whether it reveals the presence of an acute or chronic inflammation of the mucous membrane. To decide this the history and condition of the patient must be taken into account. In convalescence from pneumonia or typhoid fever the amount of pus in the urine is sometimes sufficient to cause anxiety, but as a general rule the establishment of the normal health results in the prompt disappearance of the deposit. The same conditions occur during the course of an attack of measles or diphtheria, and with rare exceptions should be regarded as a part of the elimination of the poisons of the disease, analogous to the desquamation of the skin-epithelium which often follows an acute fever. In the majority of such cases there are no symptoms which can be attributed to the pyuria, but occasionally there is a brief rise of temperature and some retention or incontinence of urine.

When this group of cases of pyuria has been set aside, there remain a considerable number in which the occurrence of pus in the urine must be regarded as a symptom of importance, and the various causes which give rise to it must be considered. The pus may be derived from any portion of the urinary tract, the pelvis of the kidney, the ureters, the bladder, or the urethra. Or it may have made its way from outside the urinary passages from an abscess, from a lumbar caries, or from a suppurating appendix. Or in female children it may come from the vulva.

In the great majority of cases in children the seat of origin is the kidney or its pelvis; and the disease is a pyelitis, a pyelonephritis, or a pyonephrosis, according to the exact condition of the organ. If the attack is an acute one the probability is largely in favour of an infection of the pelvis of the kidney: if it is chronic the more serious conditions of renal calculus and renal tuberculosis must be considered. A frank cystitis is comparatively infrequent in childhood, and when it does occur is usually associated with the classical symptoms of pain and frequency of micturition. The more common infective conditions of the whole urinary tract are often symptomless, and are entirely overlooked unless the urine is examined as part of a routine.

### The Sources of Infection.

The possible sources of infection of the urinary tract are three in number. The infective agent may travel by the blood-stream from some other focus in the body; it may ascend from the urethra and external genitals to the bladder ureters and kidneys; or it may infect the urinary organs by direct extension, that is, in all probability, by the lymphatic channels. An example of the first of these modes of infection is afforded by the acute infections of the urinary tract which occur during specific fevers, such as typhoid. Here the organism is conveyed to the kidney by the blood-stream and excreted in the urine, and in its passage may excite an acute inflammation of the pelvis of the kidney and bladder. The same explanation probably holds good for some of the more acute attacks of pyuria in the course of pneumonia and other fevers. The classical examples of ascending infections are those which follow a stricture of the urethra, and the damming back of the urine by an enlarged prostate. But it is possible that even without these mechanical obstructions an infection may spread upwards in some cases from the lower passages. For many cases it appears easier to believe that infection travels direct from the intestine to the pelvis of the kidney, though it must be admitted that proof of such transmission is wanting.

The organism which is most frequently found as the infective agent in cases of urinary infection leading to pyuria in children is the bacillus coli communis; and in the majority of cases it is present in pure culture and in enormous numbers. Less frequently there is found an organism of the proteus group, and even less often the staphylococcus pyogenes aureus. Other organisms, the typhoid bacillus, various streptococci, and rarer forms have been found; but in fully 90 per cent. of cases of infective pyelitis the active agent is the bacillus coli. Bacteriologically there are numerous varieties of this organism, and at present it does not seem possible to say that any one variety is more apt to infect the urinary passages than another. As far as diagnosis and treatment are concerned, it is sufficient to recognise that the micro-organism in question belongs to the B. coli group.

### Anatomical Effects.

The anatomical effects of an infective pyelitis of a moderate degree of intensity can easily be conjectured from a knowledge of the stages of inflammation in the case of other mucous membranes; but on one occasion these conjectures were confirmed by the appearances seen during the exploration of the pelvis of a kidney which was opened under the mistaken belief that it contained a stone. The mucous membrane was thickened and œdematous, and was streaked with dilated and tortuous capillaries. The pelvis contained a slightly turbid fluid which was swarming with bacilli; the turbidity was due partly to these, partly to desquamated epithelium and pus cells. A pelvis in such a condition is able to recover completely; but if the disturbance continues, as, for instance, in the case of a neglected pyonephrosis, it ultimately becomes an abscess cavity containing thick pus; and the pyramids of the kidney are disintegrated and disappear. In the most advanced cases the whole of the kidney substance, save perhaps a narrow ring of compressed cortex, is destroyed, and a bag of pus represents the kidney. The inflammatory process may be, and probably often is, confined to the pelvis of the kidney, or it may involve also the ureter and the bladder; and the clinical symptoms vary somewhat, according to the extent of the inflammation.

### The Symptoms of Pyelitis.

The clinical symptoms group themselves into three distinct types. (1) The acute infection, characterised by rigors, fever, and lumbar pain, with the passage of urine which contains a variable quantity of pus and the infecting organism in pure culture. (2) The subacute infection, in which, without pain or rigors, there is an unexplained fever, and wasting with anæmia, loss of appetite, and general malaise. (3) The chronic periodic infection, in which there are intervals of health, but from time to time attacks of fever, vomiting, and headache.

In all these types of the disease the urinary symptoms may be almost in abeyance, so that they pass unobserved until inquiry elicits their presence. It is, for example, not uncommon to find on examination that the urine contains considerable quantities of pus corpuscles without the patient being aware that there is anything abnormal about it. In an acute case there is, as a rule, some symptom which draws attention to the urine. Not infrequently at the onset of the attack a little blood is passed with the urine, the quantity of which may be considerably diminished. In all cases of B. coli infection that I have met with the urine has been acid in reaction, though yesterday afternoon I saw a child who *is stated* to have a bacillus coli infection, and whose urine is certainly alkaline. According to Monti, of Vienna, the specific gravity is always altered; raised in acute and much diminished in chronic cases. This observation I cannot confirm, and it is of slight importance in the diagnosis.

In all cases the urine is turbid, and the turbidity has a distinguishing characteristic. It is diminished but not removed by filtration through blotting paper. The explanation is that the filter paper removes all cells and pus corpuscles, but allows bacteria to pass freely. The deposit after the urine has stood varies considerably. In all acute and in the majority of chronic cases the deposit is very slight and may easily be overlooked. Even when centrifugalised it often forms but a very slight collection at the bottom of the tube. Microscopical examination, however, reveals its real character. In some instances the deposit may become considerable, but it never in my experience is so great in acute infective pyelitis as it is in calculous or tuberculous disease, or in cases of cystitis.

Desquamated epithelial cells from the urinary passages are generally present, but always in small amount. In a considerable percentage of cases albumin is present in the urine, at any rate in acute infections and at the beginning of an attack. It is always slight in amount, except in those cases in which there is reason to believe that the kidney tissue itself is extensively affected, as, for example, in pneumonia and scarlet fever. Crystals of uric acid and of calcium oxalate are also found occasionally; and, when the urine is strongly alkaline, crystals of triple phosphate and stellar phosphate. Casts of the kidney tubules are, in my experience, almost unknown. At most a few hyaline casts are present.

### Infections.

Of the three types of disease noted above the acute infections with marked symptoms are certainly the least common. The attack begins with a rigor, or, in young infants, with a convulsion, accompanied by a high fever, reaching 104° or even 105°. This fever as a rule is of an intermittent type, and is accompanied by sweating and abdominal and lumbar pains. The urine is often at first without obvious alteration, but in two or three days is found to contain pus and bacteria. Generally by the time the diagnosis is established the patient has improved, the fever tends to subside, and the pain to diminish, but in some cases the attack is prolonged, and the patient passes from an acute to a chronic condition. This was the case with a girl of eight years of age, who was brought to hospital ill for three weeks with acute abdominal pain, vomiting, and high fever. At the onset she also suffered from a violent diarrhœa lasting for three days. The pain was always spasmodic and was often associated with, it was alleged, swelling of the abdomen. Her temperature reached 102° on several occasions, and thrice while under observation she had retention of urine, necessitating the passage of a catheter. On examination the urine was found to contain a small quantity of albumin, a considerable quantity of pus, and a pure growth of bacillus coli communis. Her fever soon disappeared, and the condition of the urine improved with the administration of urotropine, but a small quantity of pus was still present for nearly three weeks, and the organism could be cultivated in pure growth. Eventually she recovered completely. A similar but even more acute attack in a girl of six years gave rise to pain and frequency of micturition, with the passage of albumin, blood, and pus, with fever and rigors; but in this instance the attack came to an end under the influence of urotropine in about a fortnight from its beginning.

### Subacute Infections.

The subacute infections are far more frequent and present much greater difficulties in diagnosis. Usually there is fever of an irregular character, with anæmia, loss of appetite, general malaise, and rapid wasting, without any symptoms pointing to the urinary tract. Thus a girl of ten years of age had suffered for a fortnight from what was called a bilious attack; she had vomited and had some slight fever, after which she became listless and anæmic and lost flesh rapidly. Three weeks after the bilious attack she complained on one occasion of pain on passing water. During the following month she had two or three attacks of vomiting and general disturbance, and when at the end of that time her urine was examined it was found to contain considerable quantities of pus, and a pure culture of bacillus coli communis. She took urotropine for three months, and at the end of that time her urine was clear, free from pus and bacteria, and the child was in good health and had gained weight.

Some of the subacute infections are, however, remarkably persistent. A child of thirteen months had retention of urine with slight fever and marked anæmia and wasting. The urine was clear, but on centrifugalisation was found to contain a small deposit of pus cells, and bacillus coli was grown in pure culture. A little later, in spite of treatment, the urine became offensive, and contained a considerable quantity of pus; the child began to suffer from frequency of micturition and from a good deal of pain in the bladder and urethra. With the cystoscope the bladder appeared to be normal, except for some redness at the left ureteral orifice. She was treated by various methods, but pyuria continued for ten months, when I lost sight of her.

### Chronic Periodic Pyelitis.

The most interesting cases of pyelitis, however, are the intermittent ones. The patients from time to time suffer from attacks of vomiting, headache, malaise, slight fever, and painful micturition, and recover only to relapse again a month or two later. They are interesting from several points of view; from the bacteriological, because they would appear to be instances of intermittent or periodic infection; from the clinical because of the difficulty of diagnosis unless the facts are familiar; and because when once recognised it seems to be possible to relieve them permanently in most instances.

A child of eleven and a half suffered for nearly two years from recurrent attacks of headache, vomiting, and fever, coming on at intervals of about fourteen days. Nine months previous to the date at which I first saw her she had had sharp radiating pain in the right lumbar region, and was supposed to have a stone in the kidney. Her urine at that time contained a small quantity of pus, but there was nothing else found amiss with her. Her right kidney was, however, explored and opened. No stone was found, and the kidney was described as healthy. The wound was closed, but the recurrent attacks of headache and vomiting went on as before. In October 1907 she came under my observation, and I found that the urine contained pus cells and B. coli in pure culture. She was put upon a course of urotropine and rapidly improved, the urine became free from pus and bacteria, and the attacks of vomiting ceased. Another girl, four years old, had suffered for more than a year from periodic attacks of vomiting with fever and prostration. The attacks recurred at irregular intervals of about one month, and lasted six or seven days. In the interval she seemed in good health. In one of these attacks she was found to have bacilluria, and cultivation showed a pure bacillus coli infection. She was treated for some time with small doses of urotropine, and has since remained free from the attacks of vomiting.

The resemblance of this last and the preceding cases to the condition known as cyclic vomiting will be apparent to everyone; and, though I do not suppose for a moment that all instances of cyclic vomiting are due to this cause, I think it is possible that in some such cases a urinary infection has been overlooked, especially since it is so often without any symptoms which would attract attention to the urinary organs. Cyclic vomiting is described in the text-books as more frequent in girls than in boys; brought on by fatigue and exhaustion, or excited by some temporary infection such as a slight sore throat; accompanied by fever, lassitude, headache, and some abdominal pain; and as a self-limited affection usually ending in recovery towards the end of a week. In fact, the usual text-book description of cyclic vomiting applies very well to the symptoms of intermittent chronic pyelitis.

### The Infecting Organisms.

Acute or subacute pyelitis due to an infection with bacillus coli communis is far more common than pyelitis due to other organisms. In fact in children I do not remember to have met with any other infective agent except upon one occasion when the organism was the staphylococcus pyogenes aureus. Unfortunately I cannot recover the notes of this case. In cases of secondary pyelitis, however, it is not uncommon to find other bacteria. Typhoid bacilli have been isolated from the urine so often when there has been no evidence of pyelitis that it is perhaps unjustifiable to consider their presence in the urine as having any causative influence on the pyelitis; but I can recall several instances in which organisms resembling the pneumococcus have been seen in the urine of children suffering from pneumonia, complicated by pyuria and hæmaturia.

With regard to the gonococcus, which is a frequent cause of cystitis in adults, I know of no instance in which it has caused cystitis or pyelitis in children; and this is somewhat remarkable considering the comparative frequency in hospital patients of chronic vulvo-vaginitis due to this cause. I was able a few years ago to study a small epidemic of gonorrhœal vulvo-vaginitis in the Hospital for Sick Children, and to establish the accuracy of the diagnosis by means of cultivations; but in no case was there any complication whatever, and in no case was there pain or frequency of micturition. In the United States, where several epidemics have been reported in children's hospitals, complications have occurred in many cases, most frequently conjunctivitis; but cystitis is said to be extremely rare.

### Pyelitis due to other Causes.

Apart from these cases of primary and subacute pyelitis there are the cases of calculous and tuberculous disease, and of pyuria due to infection of a congenital hydronephrosis.

With regard to tuberculous pyelitis it is, I believe, agreed that a tuberculous infection of the urinary tract is very rare in childhood: it is found post-mortem in cases of generalised tuberculous infection, but as a clinical entity it is practically unknown. In a few cases caseous tubercle occurs in one or other kidney, but it would appear from the records of the post-mortem room that in childhood it is quite likely to heal spontaneously.

Calculous pyelitis, on the other hand, is not uncommon; but can usually be easily distinguished from acute pyelitis by its history, and also by the fact that hæmaturia is in this disease a common symptom, whereas in the acute infective pyelitis it is rare.

Pyonephrosis is also a rare affection, though hydronephrosis is not very uncommon. It seems to be unusual that a hydronephrosis in childhood should become infected with pyogenic organisms. One such case, I remember, was believed to be calculous in origin. At operation, however, no stone was found either in the dilated pelvis or in the ureter. Recovery took place after prolonged drainage of the dilated pelvis.

### Pyuria Due to Phimosis.

Phimosis or a constricted meatus is sometimes the cause of a pyuria which may cause severe symptoms of general ill-health without any sign pointing to the origin of the trouble. Some time ago I saw a boy of about twelve years of age who had high fever, abdominal pain, and rapid wasting. He was admitted to the hospital, and in the course of a routine examination his urine was found to contain pus. This led to a careful examination of his urinary organs, and he was discovered to have a meatus so small that it would not admit a No. 1 catheter. The meatus was slit, and it was hoped that the condition of the urine, which was infected with bacillus coli communis, would improve. His fever fell, he became fat and well, and passed water without pain or difficulty; but in spite of treatment the bacterial infection still remained present. After a fortnight without improvement in this respect a phosphatic stone was discovered in the bladder. I mention this case in order to make three observations: First, that in the absence of all symptoms pointing to the urinary tract, it may be that there is the exciting cause of the disease; secondly, that the discovery of a constricted meatus ought to have led me to think of the possibility of the presence of a stone; and, thirdly, that the urine from first to last was acid in reaction, presumably because the infecting agent was the bacillus coli, though the calculus was phosphatic.

### Treatment.

The treatment of acute infective pyelitis is as a rule quite simple. Rest in bed is essential: even in the subacute cases the patients hardly ever improve until they are kept at rest. Attention to the condition of the bowels, and the disinfection of the urinary tract are the remaining indications. The latter is best effected by the use of urotropine or helmitol. With regard to the first it is to be noted that in some patients it has been known to cause pain and frequency of micturition, and in rare instances hæmaturia. As a rule, however, it is quite harmless if given sufficiently diluted. Accordingly I give from five to ten grains in a wineglassful of water three times a day, and by adhering to this dilution I have only once found a child who could not tolerate it. In her case there was on several occasions slight hæmaturia.

In most instances these measures are sufficient to terminate the attack quickly. But occasionally the infection persists in spite of treatment. I have not yet had an opportunity of vaccinating one of these persistent infections, but my experience of vaccination for B. coli infection in adults has not been sufficiently successful to offer much encouragement.

I have been able in adults to improve the condition of the urinary tract, even in unfavourable conditions, but I have never succeeded in banishing the infective agent. In an acute case of pyelitis in pregnancy, however, the vaccination appeared to be the cause of the disappearance of bacilli from the urine; and in any persistent infection I should certainly combine it with other measures. Recurrence of infection is certainly common in those who have not been treated, or have been treated for too short a time; but after a course of urotropine, and the establishment of urinary sterility, I have not found that recurrence takes place. I have heard, however, of one instance in which recurrences took place with such persistence that at last an exploratory operation was performed, when the tip of an inflamed appendix was found adherent to the pelvis of the kidney. In such a case it seems extraordinary that the patient should ever have had freedom from trouble.

I must apologise for treating the subject in rather a scrappy manner; but the disease is a difficult one to lecture upon because there is so little to demonstrate about it. The cases that are treated are cured. The chief interest, therefore, of the disease lies in the fact that it is one in which the diagnosis can easily be missed, especially, I believe, in the intermittent infections.

